# Artemisiae Iwayomogii Herba Protects Dopaminergic Neurons Against 1-Methyl-4-phenylpyridinium/1-methyl-4-phenyl-1,2,3,6-tetrahydropyridine Neurotoxicity in Models of Parkinson’s Disease

**DOI:** 10.3390/nu17101672

**Published:** 2025-05-14

**Authors:** Hanbyeol Lee, In Gyoung Ju, Jin Hee Kim, Yujin Choi, Seungmin Lee, Hi-Joon Park, Myung Sook Oh

**Affiliations:** 1 Department of Biomedical and Pharmaceutical Sciences, Graduate School, Kyung Hee University, Seoul 02447, Republic of Korea; 2 Department of Oriental Pharmaceutical Science and Kyung Hee East-West Pharmaceutical Research Institute, College of Pharmacy, Kyung Hee University, Seoul 02447, Republic of Korea; 3 Department of Science in Korean Medicine, College of Korean Medicine, Kyung Hee University, Seoul 02447, Republic of Korea; acufind@khu.ac.kr; 4 Acupuncture and Meridian Science Research Center (AMSRC), Kyung Hee University, Seoul 02447, Republic of Korea

**Keywords:** Parkinson’s disease, *Artemisia iwayomogi*, nuclear factor erythroid 2-related factor 2, heme oxygenase-1

## Abstract

**Background/Objectives:** Parkinson’s disease (PD) is a common neurodegenerative disease characterized by motor symptoms caused by the loss of dopaminergic neurons. While the pathophysiology of PD is still not fully understood, it is recognized that oxidative stress plays a major role in its progression. Previous studies have shown that the aerial parts of *Artemisia iwayomogi* Kitamura (AIK) possess medicinal properties, including antioxidant activity. This study aimed to investigate whether AIK can alleviate neuronal loss and motor symptoms in a PD model and to explore its therapeutic mechanisms. **Methods:** For the in vitro study, PC12 cells were treated with AIK and 1-methyl-4-phenylpyridinium (MPP^+^). For the in vivo study, C57BL/6J mice were orally administered AIK for 12 days; they received intraperitoneal injections of 1-methyl-4-phenyl-1,2,3,6-tetrahydropyridine (MPTP) for 5 consecutive days, starting on the 8th day of AIK administration. **Results:** AIK treatment to PC12 cells in the presence of MPP^+^ enhanced the phosphorylation of the protein kinase B/glycogen synthase kinase-3β signaling pathway, which is a crucial regulator of nuclear factor erythroid 2-related factor 2 (Nrf2) translocation. Additionally, AIK treatment increased cell survival and induced an antioxidant response involving heme oxygenase-1, via increasing the level of Nrf2 in the nucleus. In an MPTP-induced mouse model of PD, AIK administration activated Nrf2 in dopaminergic neurons and prevented the loss of dopaminergic neurons in the brain, which in turn alleviated motor dysfunction. **Conclusions:** Collectively, these findings suggest that AIK is a potential botanical candidate for PD treatment by protecting dopaminergic neurons through antioxidant activity.

## 1. Introduction

Parkinson’s disease (PD) is the second most common neurodegenerative disorder. Typically, PD develops after age 60, with incidence increasing with age [1]. Motor symptoms such as bradykinesia and postural instability are indicative of PD [2]. PD has key pathological features of progressive loss of dopaminergic neurons in the substantia nigra pars compacta (SNpc) and the formation of Lewy bodies [3]. The standard treatment for PD is dopamine replacement therapy (DRT), which supplements depleted dopamine levels. Levodopa (L-DOPA), a precursor of dopamine, is commonly used with dopamine agonists and monoamine oxidase B inhibitors [4]. However, DRT does not have a positive impact on disease progression, and there is currently no disease-modifying therapy available to slow or halt disease progression [5].

The pathogenesis of PD is thought to involve a multifaceted pathophysiological process, with several key pathogenic factors identified to date, including mitochondrial dysfunction, oxidative stress, and neuroinflammation [6]. Environmental and genetic variables cause mitochondrial malfunction, which raises reactive oxygen species (ROS) and worsens oxidative stress [7]. An imbalance between the capacity of the body to detoxify reactive chemicals and the generation of ROS results in oxidative stress, which seriously damages cellular structures [8].

In the PD brain, increased ROS buildup has been shown in numerous studies to be a significant factor in inflammation and dopaminergic neuronal death, which further accelerates neurodegenerative processes [9,10,11]. The SN is widely recognized to be especially susceptible to oxidative stress as it contains a high density of dopaminergic neurons [12]. Thus, oxidative stress has been recognized as a main mechanism for the death of dopaminergic neurons in PD [6]. Therefore, strategies aimed at controlling oxidative stress-induced cellular damage are crucial for preventing neurodegeneration.

*Artemisia iwayomogi* Kitamura (AIK) is a plant in the Compositae family that is distributed throughout Korea and commonly known as “Hanin-jin” or “Dowijigi” [13]. The aerial parts of AIK have been used in traditional medicine for the treatment of various liver diseases [14]. The various biological functions of AIK have been proven through numerous studies. Aqueous extracts of AIK have been shown to suppress mast-cell-mediated allergic reactions [15] and exhibit hepatoprotective activity against carbon tetrachloride-induced liver fibrosis in rats [16]. In other studies, polyphenolic extracts of AIK were shown to inhibit the expression of pro-inflammatory mediators induced by lipopolysaccharide (LPS) and to attenuate high-fat-diet-induced hypertriglyceridemia in mice [17,18]. Additionally, it has been demonstrated that the scopoletin that was separated from AIK possesses cellular antioxidant, reduction, and superoxide radical scavenging activities [19]. However, whether AIK can regulate the oxidative stress that causes neurological diseases and improve PD has yet to be investigated.

This study was conducted to investigate the potential ameliorative effects of AIK on PD in both in vitro and in vivo models. We examined how AIK treatment affects the defense mechanisms against oxidative stress in PC12 cells under 1-methyl-4-phenylpyridinium (MPP^+^)-induced PD conditions. We also assessed whether AIK administration alleviates the behavioral and histological changes in PD in an animal model induced by 1-methyl-4-phenyl-1,2,3,6-tetrahydropyridine (MPTP).

## 2. Materials and Methods

### 2.1. Materials

2,2-azinobis-(3-ethyl-benzthiazolin-6-sulphonic acid) (ABTS), 2,2-diphenyl-1-picrylhydrazyl (DPPH), MPP^+^ iodide, 3-(4,5-dimethylthiazol-2-yl)-2,5-diphenyl-2H-tetrazolium bromide (MTT), MPTP, L-DOPA, and benserazide hydrochloride were purchased from Sigma-Aldrich (St. Louis, MO, USA). Rabbit anti-protein kinase B (Akt, #9272), anti-phospho-Akt (Ser473, #4060), anti-glycogen synthase kinase-3β (GSK3β, #12456), and phospho-GSK3β (Ser9, #5558) antibodies were obtained from Cell Signaling Technology (Danvers, MA, USA). Mouse anti-proliferating cell nuclear antigen (PCNA, 610665) was purchased from BD Biosciences (Bergen County, NJ, USA). Mouse horseradish peroxidase (HRP)-conjugated β-actin antibody (sc-47778) was purchased from Santa Cruz Biotechnology (Temecula, CA, USA). Anti-rabbit HRP secondary antibody (ADI-SAB-300) and rabbit anti-heme oxygenase-1 (HO-1, ADI-SPA-895) antibody were acquired from Enzo Life Science, Inc. (Farmingdale, NY, USA). Rabbit anti-nuclear factor erythroid 2-related factor 2 (Nrf2) antibody (AB31163) was purchased from Abcam (Cambridge, UK). Rabbit anti-tyrosine hydroxylase (TH, AB152) antibody, rat anti-dopamine transporter (DAT, MAB369) antibody, and 3,3′-diaminobenzidine (DAB) were purchased from Merck Millipore (Burlington, MA, USA). Mouse anti-TH antibody (MA1-24654) and radio-immunoprecipitation assay lysis buffer (RIPA) was purchased from Thermo Fisher Scientific (Waltham, MA, USA). Biotinylated anti-rabbit immunoglobulin G (IgG) antibody (BA-1000), biotinylated anti-rat IgG antibody (BA-4000), avidin–biotin complex (ABC), anti-rabbit DyLight 594 (DI-1594), and anti-mouse DyLight 488 (DI-2488) were purchased from Vector Labs (Burlingame, CA, USA).

### 2.2. Preparation and Standardization of AIK Extract

AIK was purchased from Naemomedah (Kwangmyoungdang Medicinal Herbs, Ulsan, Republic of Korea). AIK was prepared following previously reported methods. In brief, AIK was extracted with 70% ethanol for 3 h using reflux extraction. The extract was filtered, evaporated, and lyophilized into a powder. AIK was dissolved in the vehicle before each experiment. AIK was standardized based on scopoletin and isochlorogenic acid B using ultra-performance liquid chromatography–mass spectrometry, in our previous study [20].

### 2.3. ABTS Radical Cation Scavenging Assay

One day before the experiment, 2.6 mM potassium persulfate was added to 7.4 mM ABTS solution in dark conditions. The absorbance was measured at 732 nm with a microplate reader (Sunnyvale, CA, USA) after the incubation of the pre-made ABTS solution with various concentrations of AIK for 5 min. We used an extract of *Scutellaria baicalensis* Georgi root (SB) as a positive control to assess the potency of AIK, as SB is well-known for its outstanding radical scavenging activities [21,22]. The concentration required to scavenge 50% of the ABTS cation is known as the half-maximal inhibitory concentration (IC_50_), and it was used to express the antioxidant capacity. The cation scavenging capacity of ABTS was determined using the following equation:

$$ABTS\;cation\;scavenging\;activity\;\left(\%\right)=(Control-Sample)/Control\times 100.$$

### 2.4. DPPH Radical Scavenging Assay

A microplate reader was used to detect absorbance at 517 nm after a 0.2 mM DPPH ethanolic solution was combined with different concentrations of AIK and left in the dark for 30 min. SB was used as a positive control. IC_50_ values were used to express the antioxidant capacity against the DPPH radical. The following equation was used to determine the DPPH radical scavenging capacity:

$$\mathrm{DPPH}\;\mathrm{radical}\;\mathrm{scavenging}\;\mathrm{activity}\;\left(\%\right)=\{\mathrm{Control}-\left(\mathrm{Sample}-\mathrm{Blank}\right)\}/\mathrm{Control}\times 100$$

### 2.5. Cell Culture and Drug Treatment

PC12 rat pheochromocytoma cells were cultured in Roswell Park Memorial Institute 1640 medium supplemented with 10% fetal bovine serum and 1% penicillin–streptomycin. The cells were incubated at 37 °C under a humidified atmosphere containing 5% CO_2_. The cells were seeded at a density of 3.0 × 10^5^ cells/mL in 96-well plates or 1.0 × 10^6^ cells/well in 6-well plates. After seeding, the cells were allowed to adhere for an additional 24 h. Prior to MPP^+^ treatment, the cells were pre-exposed to various concentrations of AIK for 1 h, followed by treatment with equal volumes of vehicle for both the control (CON) and MPP^+^-treated groups. Subsequently, the cells were treated with or without 1 mM MPP^+^ for 48 h for MTT assays or 4 h for Western blot analysis.

### 2.6. Measurement of Cell Viability

The MTT assay was used to evaluate cell viability. Briefly, 1 mg/mL of MTT was added to the cells and incubated for 3 h at 37 °C. Following the dimethyl sulfoxide dilution of the MTT formazan, the absorbance was measured at 570 nm using a microplate reader.

### 2.7. Western Blotting

Samples were harvested and lysed using RIPA buffer supplemented with protease and phosphatase inhibitors for whole-cell lysis. For the isolation of cytoplasmic and nuclear fractions, the collected cells were rinsed with phosphate-buffered saline (PBS) and subsequently resuspended in lysis buffer (10 mM HEPES [pH 7.9], 50 mM KCl, 1 mM EDTA, 1 mM DTT, and protease–phosphatase inhibitor cocktail). The suspension was then placed on ice for 3 min, followed by the addition of 0.075% IGEPAL CA-630. After mixing thoroughly, the samples were subjected to centrifugation at 15,000 rpm for 4 min, yielding the cytoplasmic fraction in the supernatant. The nuclear pellet was resuspended in nuclear extraction buffer (20 mM HEPES [pH 8.0], 400 mM NaCl, 1 mM EDTA, 1 mM EGTA, 1 mM DTT, and protease phosphatase inhibitor cocktail). The samples were then incubated on ice for 10 min, vortexed for 10 s, and centrifuged at 15,000 rpm for 10 min. The resulting supernatant was collected as the nuclear fraction. The resulting supernatant was used as the nuclear fraction. All protein samples were stored in a deep freezer at − 80 °C.

The Bradford protein assay was used to quantify protein concentration. After being denatured, cell lysates were separated using sodium dodecyl sulfate-polyacrylamide gel electrophoresis. Following the transfer of proteins onto polyvinylidene fluoride membranes, the membranes were blocked for 1 h using 5% bovine serum albumin. Primary antibodies were applied to the membranes overnight at 4 °C. Membranes containing bound primary antibodies were applied to secondary antibodies, and the reaction was allowed to proceed at room temperature for 1 h. The immunoreactive bands were located using the enhanced chemiluminescence reagent. The band intensity was viewed using the Image Lab (Bio-Rad Laboratories, Hercules, CA, USA) and quantified using ImageJ 1.38x (National Institutes of Health, Bethesda, MD, USA).

### 2.8. Animals and Drug Treatment

Seven-week-old male C57BL/6J mice were purchased from Daehan Biolink (Eumseong, Republic of Korea). The mice were kept in a room with consistent humidity (60  ±  10%), light/dark cycles lasting 12 h, and a temperature of 23  ±  1 °C. Food and water were freely available to the animals. Every animal study was conducted in accordance with the National Institutes of Health’s “Guide for the Care and Use of Laboratory Animals, 8th edition” (2011) and authorized by “Animal Care and Use Guidelines” of Kyung Hee University (Seoul, Republic of Korea, approval number: KHSASP-18-167; approved on 16 January 2019).

The mice were randomly divided into six groups: (1) NOR group (normal mice; intraperitoneally injected saline plus intraorally administered vehicle, *n* = 8); (2) MPTP group (intraperitoneally injected MPTP plus intraorally administered vehicle, *n* = 10); (3) MPTP + AIK 30 group (intraperitoneally injected MPTP plus intraorally administered AIK 30 mg/kg/day, *n* = 8); (4) MPTP + AIK 100 group (intraperitoneally injected MPTP plus intraorally administered AIK 100 mg/kg/day, *n* = 7); (5) MPTP + AIK 300 group (intraperitoneally injected MPTP plus intraorally administered AIK 300 mg/kg/day, *n* = 8); (6) MPTP + L-DOPA group (intraperitoneally injected MPTP plus intraorally administered L-DOPA 80 mg/kg/day with Benserazide 20 mg/kg/day, *n* = 8). The dosage of AIK was determined based on prior reports [20,23]. AIK, L-DOPA, and Benserazide were dissolved in vehicle (2% Tween 80) and were administered intraorally once per day for 12 days. MPTP was dissolved in saline and intraperitoneally injected at 30 mg/kg/day for 5 consecutive days from the 8th day of AIK administration. The time scheme of experimental procedures was shown in the Supplementary Information (Figure S1).

### 2.9. Rotarod Test

The rotarod test was performed 8 days after the last injection of MPTP. Mice were trained 1 day prior to the test. The apparatus used in the experiment had a spindle with a 3 cm diameter and was divided into five compartments. Training was performed at a constant speed of 10 rpm, and testing was performed at 11 rpm for 3 min. The time taken for each mouse to fall off the spindle for the first time and the total number of falls were measured.

### 2.10. Preparation of Brain Tissues

Tribromoethanol (312.5 mg/kg, *i.p.*) was used to administer anesthesia to each mouse 8 days after the last MPTP injection. The animals were then fixed with 4% paraformaldehyde in 0.1 M phosphate buffer after being transcardially perfused with 0.05 M PBS. Then, the brains of the mice were collected, post-fixed overnight in 4% PFA, and immersed in a solution containing 30% sucrose in PBS until they were dehydrated at 4 °C. Then, 25 µm thick coronal sections were created using a cryostat (Leica Microsystems Inc., Nussloch, Germany) and kept at 4 °C in a cryoprotectant solution (25% ethylene glycol, 25% glycerol, 0.05 M phosphate buffer).

### 2.11. Immunohistochemistry and Immunofluorescence

Brain sections were rinsed with PBS and treated with 1% hydrogen peroxide in 0.05 M PBS for 15 min. Following incubation, the sections underwent PBS wash before being exposed to anti-TH or anti-DAT antibody, which were dissolved in 0.3% Triton X-100 and incubated overnight at 4 °C. Next, the sections were treated with biotinylated anti-rabbit or anti-rat IgG antibody for 1 h, followed by ABC solution for 1 h at room temperature. Then, DAB was added to 0.05 M Tris buffer to improve staining visibility. Following several PBS washes, histomount media were used to mount the sections. Images were obtained with an optical bright-field microscope (Olympus Microscope System BX51; Olympus, Tokyo, Japan). Optical densities of TH- and DAT-positive areas were measured using the Image J software [National Institutes of Health (Bethesda, MD, USA)]. TH-immunopositive cells were quantified by stereological quantification. Data are shown as percentages of the NOR group values.

In order to obtain fluorescence images, brain sections were placed in anti-Nrf2 antibody overnight at 4 °C and then incubated with anti-rabbit Alexa 594 for 1 h at room temperature. The sections were rinsed with PBS, treated with anti-TH antibody for a whole night at 4 °C, then incubated with anti-mouse DyLight 488 for 1 h at room temperature. The sections were stained for 1 h with 4′,6-diamidino-2-phenylindole (DAPI) and then placed on an anti-fade fluorescent mounting medium. A Nanoscope Systems K1-Fluo confocal microscope (Nanoscope Systems, Daejeon, Republic of Korea) was used to take pictures.

### 2.12. Statistical Analysis

To calculate all statistical parameters, GraphPad Prism 8.0 software (Graphpad Software, San Diego, CA, USA) was used. The results were presented as the mean ± standard error of the mean (S.E.M.) and examined using one-way analysis of variance (ANOVA) followed by Dunnett’s post hoc test. *p* values below 0.05 were regarded as statistically significant differences.

## 3. Results

### 3.1. Effects of AIK on Free Radical Scavenging Activities

**Figure 1:**
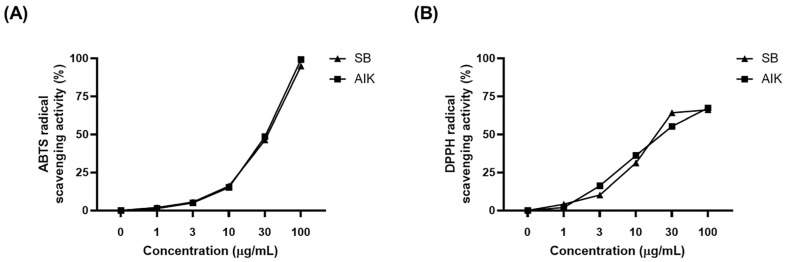
AIK possesses free radical scavenging activity. (**A**) ABTS radical cation and (**B**) DPPH free radical scavenging activities at concentrations of 1–100 μg/mL.

The antioxidant activity of AIK was assessed by measuring its capacity to scavenge ABTS and DPPH radicals. In these experiments, SB, which has been reported to have potent antioxidant activity, was used as a positive control [22]. AIK exhibited dose-dependent scavenging activity against radicals in both assays. AIK showed IC_50_ values (μg/mL) of 32.95 and 25.14 for ABTS and DPPH radicals, respectively, which were comparable to those of the SB extract (Figure 1).

### 3.2. Effects of AIK on MPP^+^-Induced Cytotoxicity in PC12 Cells

**Figure 2:**
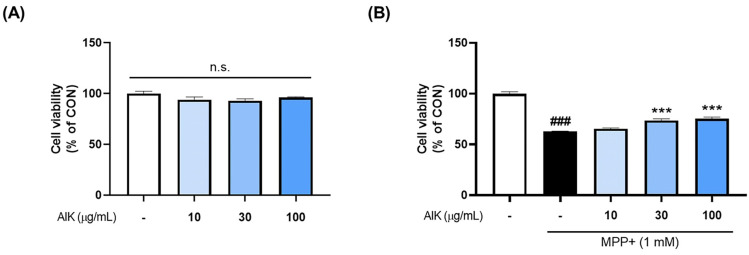
AIK treatment protects PC12 cells from MPP^+^-induced cytotoxicity. (**A**) After PC12 cells were stabilized, they were treated with various concentrations of AIK, and cell viability was measured 48 h later. (**B**) Various concentrations of AIK were treated for 1 h and then incubated with 1 mM MPP^+^ for an additional 48 h. Values are indicated as the mean  ±  S.E.M. Data were analyzed by one-way ANOVA, followed by Dunnett’s multiple-comparison test. ### *p* < 0.001 compared to the CON group; *** *p* < 0.001 compared to the MPP^+^ treated group. n.s.; not significant.

To determine the protective effect of AIK, we assessed the survival of PC12 cells after AIK treatment, with or without MPP^+^ treatment. The cells were treated with different concentrations of AIK for 1 h before being exposed to MPP^+^ for 48 h, after which we assessed the viability of the cells. The results showed that AIK alone had no significant effect on cell viability within the 10–100 μg/mL range. Treatment with 1 mM MPP^+^ led to a considerable decrease in cell viability when compared to the CON group; however, pre-treatment with AIK was shown to significantly increase cell viability compared to the MPP^+^-treated group (Figure 2).

### 3.3. Effects of AIK on Akt/GSK3β/Nrf2 Signaling Pathway in PC12 Cells

**Figure 3:**
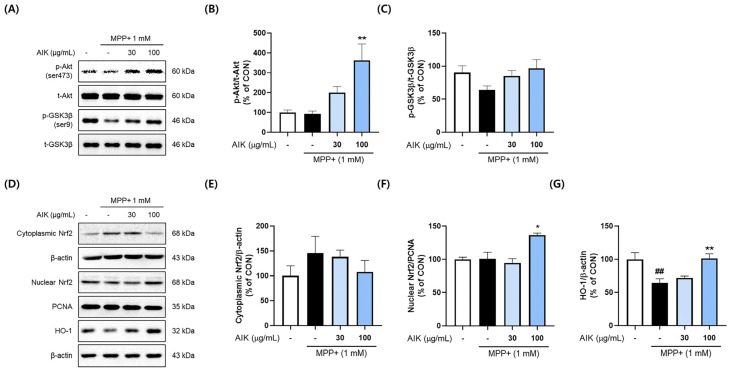
AIK treatment stimulates the Akt/GSK3β/Nrf2 signaling pathway to induce the expression of HO-1 in PC12 cells. Cells were treated with AIK at concentrations of 30 and 100 μg/mL and then incubated with 1 mM of MPP^+^ for 4 h. (**A**) Representative band images and the quantifications of (**B**) p-Akt normalized to t-Akt and (**C**) GSK3β normalized to t-GSK3β. (**D**) Representative band images and quantifications of (**E**) cytoplasmic Nrf2, (**F**) nuclear Nrf2, and (**G**) HO-1. Cytoplasmic Nrf2 and HO-1 were normalized to β-actin, and nuclear Nrf2 was normalized to PCNA. Values are indicated as the mean  ±  S.E.M. Data were analyzed by one-way ANOVA, followed by Dunnett’s multiple-comparison test. ## *p* < 0.01 compared to the control group; ** *p* < 0.01 and * *p* < 0.05 compared to the MPP^+^-treated group.

To explore how AIK protected PC12 cells, we analyzed whether it affected antioxidant factors. The phosphorylation of Akt/GSK3β is known to facilitate Nrf2 translocation [24]. To investigate whether AIK treatment affected the activation of the Akt/GSK3β signaling pathway, Western blot analysis was performed. Although the CON and MPP^+^-treated group did not differ significantly, AIK pre-treatment markedly raised the phosphorylation levels of Akt (ser473) in comparison to the MPP^+^-treatment group (Figure 3B). Treatment with MPP^+^ reduced the GSK3β (ser9) phosphorylation, whereas treatment with AIK tended to increase levels (Figure 3C).

We then measured cytoplasmic and nuclear Nrf2 expression levels to assess the effect of AIK on the translocation of Nrf2. The AIK-treated group exhibited markedly elevated nuclear Nrf2 protein levels, whereas cytoplasmic Nrf2 levels were notably lower in the AIK-treated group compared to the MPP^+^-treated group (Figure 3E,F). The movement of Nrf2 from the cytoplasm to the nucleus plays a role in regulating HO-1 expression [25]. The MPP^+^-treated group showed a marked decrease in HO-1 protein expression relative to the CON group, whereas the HO-1 protein levels in the AIK-treated group were considerably restored to levels similar to those of the CON group (Figure 3G).

### 3.4. Effects of AIK on the Nuclear Translocation of Nrf2 in Dopaminergic Neurons in Mice with MPTP-Induced PD

**Figure 4:**
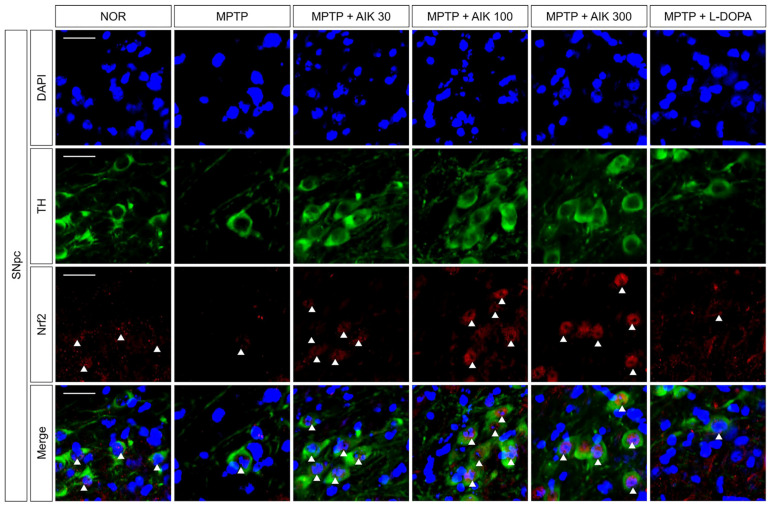
AIK administration induces the translocation of Nrf2 to the nucleus of dopaminergic neurons in the SNpc of MPTP-induced PD mice. Representative images of Nrf2 translocated to the nucleus of TH-positive dopaminergic neurons in SNpc are shown. Scale bar = 50 μm (*n* = 7–10 per group). The presence of Nrf2 in the nucleus of dopaminergic neurons was indicated by white arrows.

We used double immunofluorescence staining against TH and Nrf2 to examine whether AIK affected Nrf2 translocation in the SNpc of mice with PD. There was no discernible difference in Nrf2 translocation between the MPTP and NOR groups. In contrast to the MPTP group, Nrf2 translocation was increased in the AIK-treated groups. The MPTP + L-DOPA group and the MPTP group did not differ in their Nrf2 translocation (Figure 4).

### 3.5. Effects of AIK on Dopaminergic Neuronal Loss in Mice with MPTP-Induced PD

**Figure 5:**
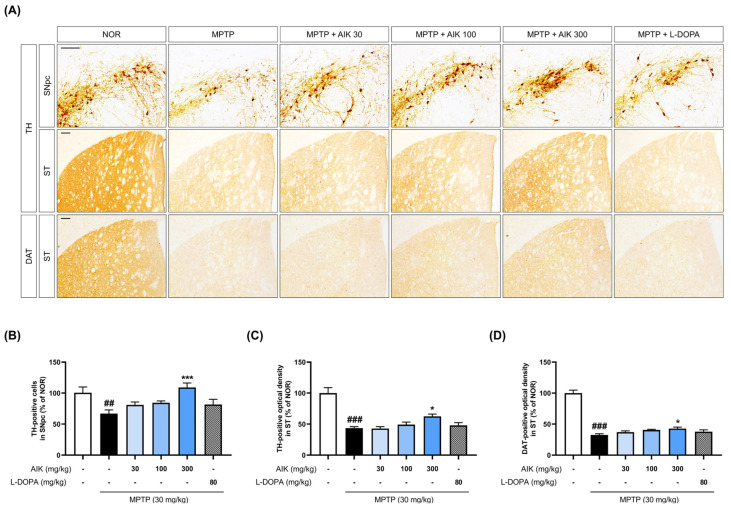
AIK administration protects dopaminergic neurons in the SNpc and ST of MPTP-induced PD mouse brain. (**A**) Dopaminergic neurons were visualized using immunohistochemistry. (**B**) The numbers of TH-positive cells in the SNpc were counted, and the optical density of (**C**) TH- and (**D**) DAT-positive fibers was measured in the ST. Scale bar = 200 μm (*n* = 7–10 per group). Values are indicated as the mean  ±  S.E.M. Data were analyzed by one-way ANOVA, followed by Dunnett’s multiple-comparison test. ## *p* < 0.01 and ### *p* < 0.001 compared to the NOR group; * *p* < 0.05 and *** *p* < 0.001 compared to the MPTP group.

Immunohistochemistry for TH and DAT, which are markers of dopaminergic neurons, was performed in order to evaluate the protective effects of AIK on nigrostriatal dopaminergic neuronal loss. When TH-positive cells in the SNpc were quantified, the MPTP group had a significantly lower number of dopaminergic neurons than the NOR group. The TH-positive cell count was significantly higher in the MPTP + AIK 300 group than in the MPTP group. Additionally, the striatum (ST) of the MPTP group contained noticeably fewer TH- and DAT-positive fibers than those of the NOR group. However, compared to the MPTP group, we discovered that the MPTP + AIK 300 group had markedly more TH- and DAT-positive fibers. Treatment with L-DOPA had no effect on the survival of dopaminergic cells (Figure 5).

### 3.6. Effects of AIK on Behavioral Impairment in Mice with MPTP-Induced PD

**Figure 6:**
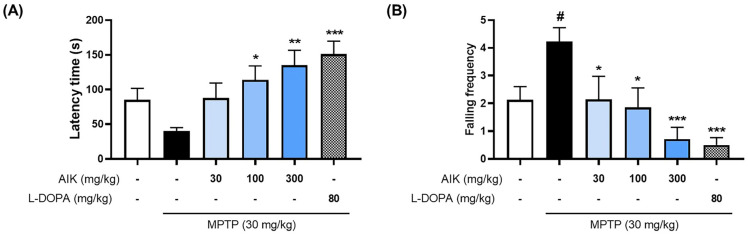
AIK administration ameliorates motor deficits in MPTP-induced PD mice. (**A**) Latency time to the first fall and (**B**) the frequency of falls during 3 min in the rotarod test were recorded (*n* = 7–10 per group). Values are indicated as the mean  ±  S.E.M. Data were analyzed by one-way ANOVA, followed by Dunnett’s multiple-comparison test. # *p* < 0.05 compared to the NOR group; * *p* < 0.05, ** *p* < 0.01, *** *p* < 0.001 compared to the MPTP group.

The rotarod test was used to determine whether AIK improves motor dysfunction in mice with MPTP-induced PD. The MPTP group showed shorter latency times until falling, compared to the NOR group, and recorded significantly higher frequencies of falls. However, the time spent on the rotarod spindle was relatively longer in the AIK-treated group than the MPTP group, and the falling frequency was significantly lower. Latency times were significantly increased, while the number of falls was significantly reduced in the MPTP + L-DOPA group compared with the MPTP group (Figure 6).

## 4. Discussion

This study demonstrated that AIK had a mitigating effect on dopaminergic neuronal damage and motor impairments. AIK exhibited potent ABTS and DPPH radical scavenging activities. Furthermore, experiments in PC12 cells stimulated with MPP^+^ showed that pre-treatment with AIK prevented cell death and significantly upregulated HO-1 levels through the activation of the Akt/GSK3β /Nrf2 pathway. In addition, the administration of AIK in mice with MPTP-induced PD activated the Nrf2 system in dopaminergic neurons, thereby protecting these neurons and subsequently improving behavioral impairments.

MPTP is a widely used neurotoxin that induces oxidative stress, leading to dopaminergic neuronal degeneration in the brains of animals. Upon injection into mice, MPTP crosses the blood–brain barrier and is metabolized to MPP^+^, the active neurotoxic form, within astrocytes [26]. The overaccumulation of MPP^+^ in the synaptic vesicles of dopaminergic neurons inhibits mitochondrial electron transport chain complex I, resulting in ROS generation and ultimately causing cellular damage and apoptosis. Simultaneously, it activates microglia, triggering the release of pro-inflammatory cytokines [27]. In this study, we utilized MPP^+^ in vitro, which directly induces neurotoxicity in neuronal cells, and demonstrated that AIK is a potential neuroprotective agent. The results obtained from our in vivo study using MPTP as a toxin confirmed the therapeutic efficacy of AIK in combating PD, as evidenced by the restoration of motor function and neuronal survival.

Oxidative stress, primarily caused by the accumulation of ROS, is one of the major pathogenic mechanisms in MPTP-induced PD, and it is counteracted by defense mechanisms activated through the Nrf2-mediated pathway [28]. The regulation of ROS is primarily mediated by Nrf2 and its downstream mediator, HO-1. As a key regulatory protein, Nrf2 controls how cells react to environmental stimuli that cause oxidative stress. By stimulating the expression of several antioxidant enzymes, such as HO-1, which controls ROS levels to avoid excessive accumulation, Nrf2 shields cells from harm caused by ROS [29,30]. Oxidative damage is closely linked to the activation of cell death signaling pathways. The activation of the Akt/GSK3β signaling pathway under oxidative conditions is known to promote the nuclear translocation of Nrf2, thereby contributing to the regulation of neuronal survival or death [31]. Kelch-like ECH-associated protein 1 (Keap1) and Nrf2 coexist in the cytoplasm under normal physiological settings. On the other hand, Nrf2 separates from Keap1 during stimulation and binds to DNA sites in the nucleus called antioxidant response elements (AREs) [32]. Nrf2 translocated to the nucleus is converted into a heterodimer form with the Maf protein, and this heterodimer binds to the antioxidant response element to induce the expression of genes that protect the cell. Nrf2-mediated expression of antioxidant factors restores the redox imbalance and induces a response to cellular damage and inflammation [33,34].

HO-1 is a key antioxidant enzyme induced by this process. It is upregulated upon exposure to oxidative stimuli and protects neuronal cells through its role in heme group degradation and the subsequent yield of biliverdin, carbon monoxide, and free iron. In addition to reducing oxidative damage, the compounds generated by HO-1 have been shown to have anti-inflammatory properties by scavenging ROS [35]. Biliverdin reductase (BVR) transforms the biliverdin generated during heme breakdown by HO-1 into bilirubin, a potent antioxidant. Continuous ROS clearance is made possible by bilirubin’s 1:1 stoichiometry of scavenging ROS, oxidizing to biliverdin, which is then reduced to bilirubin by BVR [36,37]. Extensive studies have demonstrated that HO-1 plays a role in protecting neuronal cells from oxidative stress. Wang et al. reported that the Nrf2/HO-1 pathway mediates neuroprotective effects through the mitochondrial apoptotic pathway and the alleviation of neuroinflammation [38]. Jin et al. demonstrated that *Acanthopanax senticosus* (Rupr. & Maxim.) Harms inhibited neuroinflammation and protected neuronal cells against glutamate-induced toxicity through the regulation of HO-1 signaling [39]. Morroni et al. reported that caffeic acid phenethyl ester attenuated amyloid-β peptide-induced neuronal apoptosis and neuroinflammation through the activation of the Nrf2/HO-1 pathway in a mouse model of Alzheimer’s disease [40]. In a similar context, we found that AIK activated the Akt/Nrf2 pathway and upregulated the expression of HO-1 in PC12 cells stimulated with MPP^+^, suggesting that AIK confers protection against oxidative stress.

The intricate relationship between inflammation and oxidative stress has been demonstrated in numerous studies. ROS released from damaged mitochondria exacerbate oxidative stress by activating various inflammatory pathways [41]. Specifically, inflammatory responses are induced by activated astrocytes and microglia, leading to neurodegeneration and cell death in PD [42]. In our previous study, we confirmed the anti-inflammatory effects of AIK on LPS-induced inflammation [20]. AIK inhibited nuclear factor kappa-light-chain-enhancer of activated B cells and mitogen-activated protein kinase-mediated pro-inflammatory pathways in LPS-stimulated BV2 microglial cells and suppressed the activation of microglia and the nucleotide-binding oligomerization domain, leucine-rich repeat, and pyrin domain-containing protein 3 inflammasome in LPS-injected mice [20]. Another study discovered that scopoletin, a major component of AIK, enhances antioxidant signaling via increased nuclear translocation of Nrf2 and offers neuroprotection against rotenone-induced neurotoxicity in SH-SY5Y cells [43]. Additional findings from this investigation demonstrated that AIK reduced neuroinflammation in the MPTP-induced PD mice’s SN and ST (Figures S2 and S3). Taken together, data from previous studies and the current study suggest that AIK is a promising therapeutic agent that modulates neuroinflammation and protects neuronal cells, leading to integrative and effective treatment of PD.

## 5. Conclusions

In conclusion, we demonstrated that AIK exerted neuroprotective effects on dopaminergic neurons by promoting antioxidant signaling and ameliorating motor impairment and neuroinflammation. These results suggest that AIK is a promising therapeutic approach to prevent or treat the progression of PD.

## Data Availability

Data are contained within the article or Supplementary Material.

## Supplementary Materials

The following supporting information can be downloaded at: https://www.mdpi.com/article/10.3390/nu17101672/s1. Figure S1. The time scheme of experimental procedures. Figure S2: AIK administration inhibits the activation of microglia in the brain of MPTP-induced mice. Figure S3: AIK administration inhibits the activation of astrocytes in the brain of MPTP-induced PD mice.

## Abbreviations

ABC	avidin–biotin complex
ABTS	2,2-azinobis-(3-ethyl-benzthiazolin-6-sulphonic acid)
AIK	aerial parts of *Artemisia iwayomogi* Kitamura
Akt	protein kinase B
ANOVA	analysis of variance
AREs	antioxidant response elements
BVR	biliverdin reductase
CON	control
DAB	3,3′-diaminobenzidine
DAPI	4′,6-diamidino-2-phenylindole
DAT	dopamine transporter
DPPH	2,2-diphenyl-1-picrylhydrazyl
DRT	dopamine replacement therapy
GSK3β	glycogen synthase kinase-3β
HO-1	heme oxygenase-1
HRP	horseradish peroxidase
IC_50_ values	half-maximal inhibitory concentration
IgG	immunoglobulin G
Keap1	Kelch-like ECH-associated protein 1
L-DOPA	levodopa
LPS	lipopolysaccharide
MPP+	1-methyl-4-phenylpyridinium
MPTP	1-methyl-4-phenyl-1,2,3,6-tetrahydropyridine
MTT	3-(4,5-dimethylthiazol-2-yl)-2,5-diphenyl-2H-tetrazolium bromide
Nrf2	nuclear factor erythroid 2-related factor 2
PBS	phosphate-buffered saline
PCNA	proliferating cell nuclear antigen
PD RIPA	Parkinson’s disease radio-immunoprecipitation assay lysis buffer
ROS	reactive oxygen species
SB	*Scutellaria baicalensis* Georgi root
S.E.M	standard error of the mean
SN	substantia nigra
SNpc	substantia nigra pars compacta
ST	striatum
TH	tyrosine hydroxylase
