# Supplementary material for: Artemisiae Iwayomogii Herba Protects Dopaminergic Neurons Against 1-Methyl-4-phenylpyridinium/1-methyl-4-phenyl-1,2,3,6-tetrahydropyridine Neurotoxicity in Models of Parkinson’s Disease
Source: Nutrients. 2025 May 14;17(10):1672. doi: 10.3390/nu17101672 (PMC12114580; doi:10.3390/nu17101672)
Supplement: Supplementary file 1 [file nutrients-17-01672-s001.zip › nutrients-3509903-supplementary.pdf]

< Supplementary Information>

Artemisiae iwayomogii Herba Protects Dopaminergic Neurons Against 1-Methyl-4-phenylpyridinium/1-methyl-4-phenyl-1,2,3,6-tetrahydropyridine Neurotoxicity in Models of Parkinson's Disease

Hanbyeol Lee[1, †], In Gyoung Ju[2, †], Jin Hee Kim[1], Yujin Choi[1], Seungmin Lee[1], Hi-Joon Park[3, 4], Myung Sook Oh[1, 2, *]

[1] Department of Biomedical and Pharmaceutical Sciences, Graduate School, Kyung Hee University, Seoul, Republic of Korea

[2] Department of Oriental Pharmaceutical Science and Kyung Hee East-West Pharmaceutical Research Institute, College of Pharmacy, Kyung Hee University, Seoul, Republic of Korea

[3] Department of Science in Korean Medicine, College of Korean Medicine, Kyung Hee University, Seoul, Republic of Korea

[4] Acupuncture and Meridian Science Research Center~(AMSRC), Kyung Hee University, Seoul, Republic of Korea

†These authors contributed equally to this work.

*Corresponding author

Myung Sook Oh

Department of Biomedical and Pharmaceutical Sciences, Graduate school, and Department of Oriental Pharmaceutical Science, College of Pharmacy, Kyung Hee University, Seoul 02447, Republic of Korea

Tel.: +82 2 961 9436; Fax: +82 2 963 9436.
E-mail address: msohok@khu.ac.kr

Material & Method

Materials

Rabbit anti-ionized calcium-binding adapter molecule-1 (Iba-1, 019-19741) antibody was purchased from Fujifilm Wako (Chuo-Ku, Osaka, Japan). Rabbit anti-Glial fibrillary acidic protein (GFAP, PA3-16727) antibody and mouse anti-TH antibody (MA1-24654) were purchased from Thermo Fisher Scientific (Waltham, MA, USA). Biotinylated anti-rabbit immunoglobulin G (IgG) antibody (BA-1000), avidin–biotin complex (ABC), anti-rabbit DyLight 594 (DI-1594) and anti-mouse DyLight 488 (DI-2488) were purchased from Vector Labs (Burlingame, CA, United States).

Preparation of Brain Tissue

On day 8th day after the last MPTP injection, every mouse was anesthetized with tribromoethanol (312.5 mg/kg, *i.p.*). The mice were perfused transcardially with 0.05 M PBS, and then fixed with pre-chilled 4% PFA in 0.1 M phosphate buffer (PB). Brains were dissected from the skull, post-fixed overnight in buffered 4% PFA, and incubated in 0.05 M PBS containing 30% sucrose solution until dehydration at 4 °C. Serial 25-μm thick coronal sections were prepared by a cryostat (Leica Camera Wetzlar, Germany) and then stored in cryoprotectant (25% ethylene glycol, 25% glycerol, 0.05 M PB) at 4 °C.

Immunohistochemistry

Brain sections were rinsed in PBS and incubated with 1% H_2O_2 in 0.05 M PBS for 15 min. After incubation, the sections were washed with PBS again and then incubated with anti-Iba-1 antibody (1:1000) or anti-GFAP antibody (1:2000) diluted in 0.3% Triton X-100 overnight at 4 °C. Subsequently, they were incubated with biotinylated anti-rabbit IgG antibody (1:500) for 1 h and ABC solution (1:100) for 1 h at room temperature. To visualize the staining in each section, DAB in 0.05 M tris–buffer was utilized. After several rinses with PBS, the sections were mounted with histomount medium. The images were captured by using an optical bright-field microscope (Olympus Microscope System BX51; Olympus, Tokyo, Jappan).

For fluorescence images, the brain sections were incubated with anti-Iba-1 antibody (1:1000) or anti-GFAP antibody (1:2000) overnight at 4°C and then with anti-rabbit Alexa 594 (1:500) for 1 h at room temperature. After washing with PBS, the sections were incubated with anti-TH antibody (1:1000) overnight 4°C and then anti-mouse DyLight 488 (1:500) for 1 h at room temperature. The

sections were washed with PBS, and mounted with anti-fade fluorescent mounting medium. The images were captured using a K1-Fluo confocal microscope (Nanoscope Systems, Daejeon, Republic of Korea).

Immunopositive area percentage of GFAP were measured using the Image J software [National Institutes of Health (Bethesda, MD, USA)]. Iba-1 immunopositive cells were quantified by stereological analyses.

Statistical analysis

All statistical parameters were calculated using GraphPad Prism 8.0 software (Graphpad Software, San Diego, CA, USA). Values were expressed as the mean ± standard error of the mean (S.E.M.) and analyzed using one-way analysis of variance (ANOVA), followed by Dunnett’s post hoc test. Differences with a p value less than 0.05 were considered statistically significant.

Results

Effects of AIK on neuroinflammation in mice with MPTP-induced PD

We analyzed gliosis in the brains of mice with MPTP-induced PD to investigate the effects of AIK on neuroinflammation. First, to explore the effects of AIK on microglial activation in the substantia nigra (SN), the expression of Iba-1, a marker of microglia, was analyzed by double immunofluorescence staining along with TH. A significant increase in the number of Iba-1 positive cells was observed in the MPTP group compared to the NOR group. After treatment with AIK, there was a significant, dose-dependent decrease in Iba-1-positive cell numbers. In the ST, similar to the SN, the number of Iba-1-positive cells were found to be high in the MPTP group and markedly decreased in the AIK-treated group. Iba-1 levels in the MPTP + L-DOPA group, which served as the positive control, showed no significant changes in either the SN or the ST regions compared to its levels in the MPTP group (Figure S2).

To analyze reactive astrocytes, GFAP was used as a marker of astrocytes. Similar to the findings for Iba-1, the area of GFAP-positive cells markedly increased in the SN of the MPTP group, whereas AIK treatment significantly decreased GFAP levels in a dose-dependent manner. Furthermore, a significant increase in GFAP levels was observed in the ST; however, AIK treatment successfully reduced these levels (Figure S3).

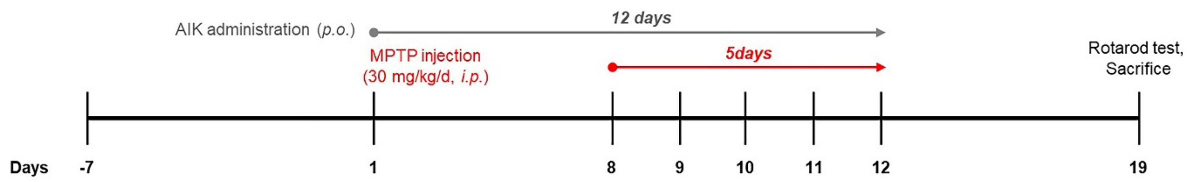

Figure S1. The time scheme of experimental procedures. *p.o.*; per oral, *i.p.*; intraperitoneal, AIK; *Artemisia iwayomogi* Kitamura, MPTP; 1-methyl-4-phenyl-1,2,3,6-tetrahydropyridine

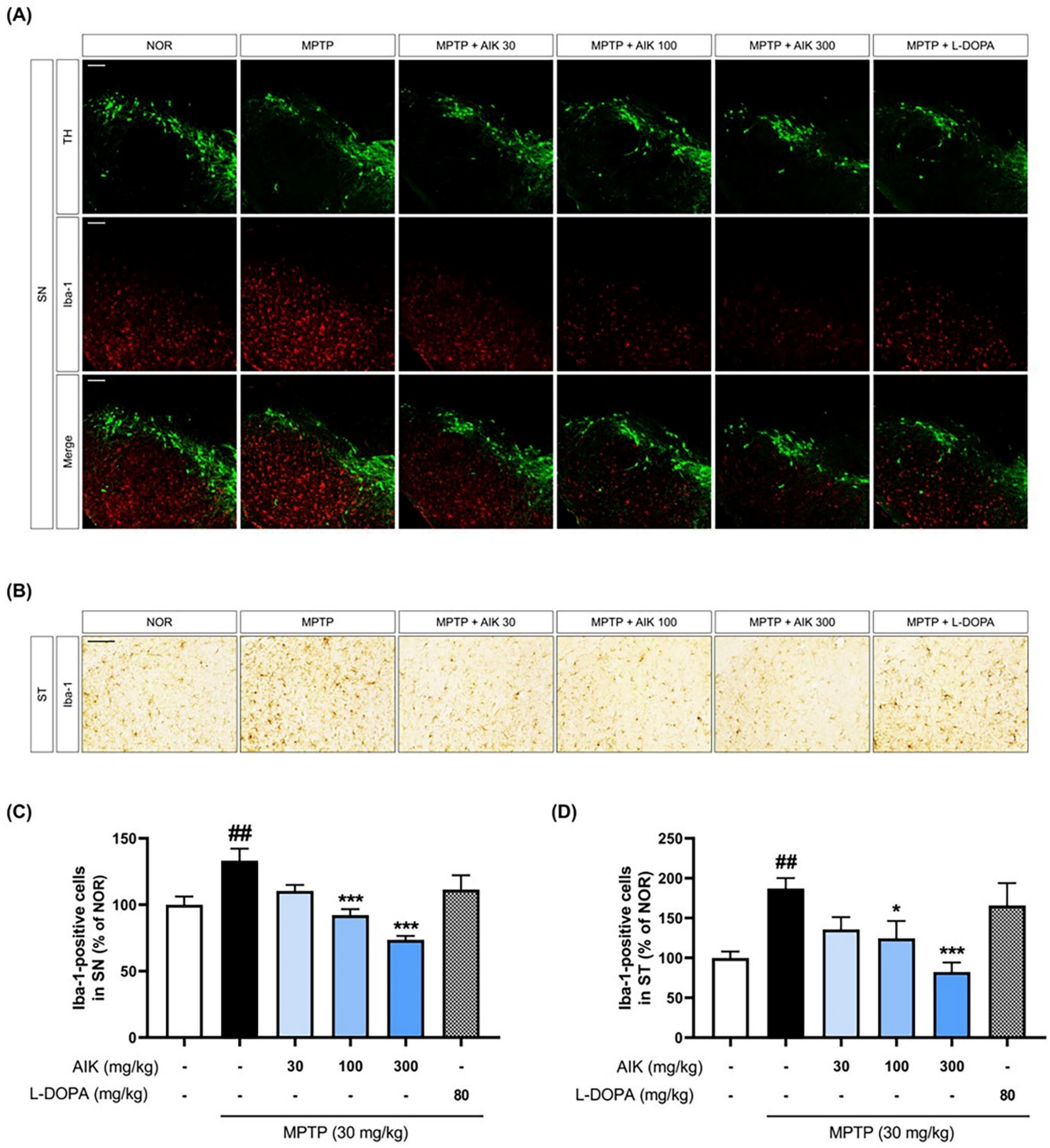

Figure S2. AIK administration inhibits the activation of microglia in the brain of MPTP-induced mice

Representative images and the quantifications of Iba-1-positive cells in the (A, C) SN and (B, D) ST. Scale bar = 100 μm. Values are indicated as the mean ± S.E.M. Data were analyzed by one-way ANOVA, followed by Dunnett's multiple comparisons test. ## p<0.01 compared to the NOR group; *p<0.05 and ***p<0.001 compared to the MPTP group

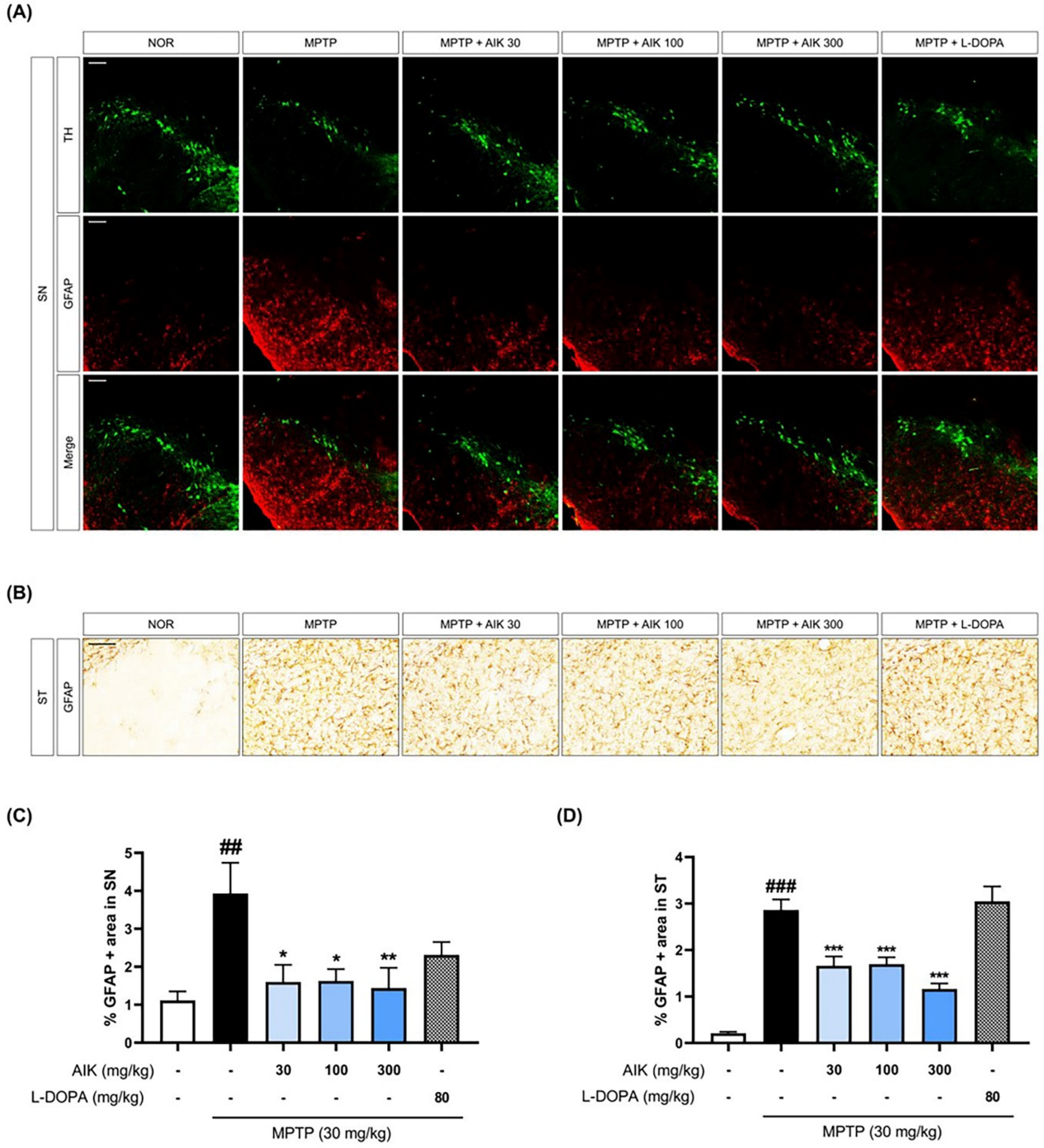

Figure S3. AIK administration inhibits the activation of astrocytes in the brain of MPTP-induced PD mice

Representative images and the quantifications of GFAP-positive area in the (A, C) SN and (B, D) ST. Scale bar = 100 μm. Values are indicated as the mean ± S.E.M. Data were analyzed by one-way ANOVA, followed by Dunnett's multiple comparisons test. ##*p*<0.01 and ###*p*<0.001 compared to the NOR group; **p*<0.05, **p*<0.01, and ****p*<0.001 compared to the MPTP group
